# High-flow nasal cannula versus non-invasive ventilation for acute exacerbations of chronic obstructive pulmonary disease with acute-moderate hypercapnic respiratory failure: a retrospective study

**DOI:** 10.3389/fmed.2025.1582749

**Published:** 2025-06-18

**Authors:** Nicolás Colaianni-Alfonso, Ada Toledo, Guillermo Montiel, Cristian Deana, Luigi Vetrugno, Mauro Castro-Sayat

**Affiliations:** ^1^Respiratory Intermediate Care Unit, Hospital Juan A. Fernández, Buenos Aires, Argentina; ^2^Department of Anesthesia and Intensive Care, Health Integrated Agency of Friuli Centrale, Udine, Italy; ^3^Department of Medical, Oral and Biotechnological Sciences, University of Chieti-Pescara, Chieti, Italy

**Keywords:** acute hypercapnic respiratory failure, COPD, non-invasive ventilation, high-flow nasal cannula, non-invasive respiratory support (NIRS)

## Abstract

**Background:**

Acute exacerbations of chronic obstructive pulmonary disease (AECOPD) frequently present with acute hypercapnic respiratory failure (AHRF). While non-invasive ventilation (NIV) remains the fist-line therapy, high-flow nasal cannula (HFNC) offers a potential alternative.

**Methods:**

This retrospective cohort study compared the clinical effectiveness and safety of HFNC versus NIV as initial respiratory support in 100 consecutive patients with AECOPD and AHRF (PaCO2 > 45 mmHg, pH 7.25–7.35). Patients were categorized into HFNC and NIV groups based on the respiratory support initiated within the first 2 h of admission. The primary outcome was treatment failure, defined as intubation, switch from one non-invasive respiratory support to another or death under NIRS. Secondary outcomes included respiratory rate (RR), arterial blood gas parameters, length of stay, and duration of respiratory support.

**Results:**

Treatment failure rates were comparable between the HFNC (32%) and NIV (35%) groups (*p* = 0.72). However, reasons for treatment escalation differed significantly. NIV failure was largely due to intolerance, while HFNC failure was associated with worsening respiratory distress or hypercapnia. NIV demonstrated superior early improvements in RR and PaCO2 compared to HFNC. No statistically significant differences were found in length of stay or 28-day mortality.

**Conclusion:**

This study suggests similar overall treatment success rates for HFNC and NIV in AECOPD with AHRF. However, NIV appears more effective in achieving early respiratory improvements, whereas HFNC offers superior tolerability. Further large-scale, prospective, randomized controlled trials are warranted to definitively establish optimal respiratory support strategies for this patient population.

## Introduction

Chronic obstructive pulmonary disease (COPD) is a prevalent chronic respiratory condition that remains a major cause of morbidity and mortality worldwide. Acute exacerbations of COPD (AECOPD) are the leading cause of death. Non-invasive ventilation (NIV) is recommended as the standard therapy for cases of AECOPD complicated by moderate hypercapnic acute respiratory failure (AHRF) (1). However, several factors may contribute to NIV failure, including discomfort associated with the interface, patient–ventilator asynchrony, excessive airway secretions, disease severity, and the expertise of the healthcare team (2–4). High-flow nasal cannula (HFNC) oxygen therapy has demonstrated both physiological and clinical benefits in patients with COPD (5). It generates a distending pressure, producing a positive end-expiratory pressure (PEEP) effect that may counteract intrinsic PEEP (6). Additionally, it facilitates the washout of nasopharyngeal dead space, thereby optimizing ventilatory efficiency and promoting carbon dioxide clearance (7, 8). HFNC also reduces inspiratory resistance by delivering adequate flow and humidified (9), warmed gases, helping prevent bronchoconstriction triggered by dry air (10). In addition, it may improve mucociliary clearance (11) and reduce diaphragmatic workload in a manner comparable to non-invasive ventilation (NIV) (12). Interestingly, during HFNC or NIV therapy, patients may simultaneously receive repeated doses of inhaled bronchodilator using a vibrating mesh nebuliser (VMN) placed at the inlet of the humidifier without discontinuing HFNC treatment or placed at Y-connection during NIV (13–15).

Recent evidence suggests that HFNC may play a positive role in the management of patients with AECOPD. Non-randomized studies have indicated that HFNC is comparable to NIV in preventing endotracheal intubation (ETI) in patients with mild-to-moderate AECOPD and respiratory acidosis, with similar failure rates. However, HFNC has been associated with greater patient comfort and fewer complications compared to NIV (16, 17). A recent multicenter randomized non-inferiority trial by Cortegiani et al. (18) demonstrated that HFNC was statistically non-inferior to NIV as an initial ventilatory support strategy for reducing PaCO₂ after 2 h of treatment in patients with mild-to-moderate AECOPD, using a non-inferiority margin of 10 mmHg. However, 32% of patients receiving HFNC required escalation to NIV within 6 h (18). A more recent randomized, open-label, non-inferiority trial conducted by Tan et al. aimed to compare treatment failure rates between HFNC and NIV in this patient population. The study concluded that HFNC did not meet non-inferiority criteria compared to NIV and was associated with a higher incidence of treatment failure when used as the initial respiratory support in patients with AECOPD and acute moderate hypercapnic respiratory failure (19). The aim of our study is to compare the use of HFNC versus NIV as first-line treatment in AECOPD and to identify the failure rate, defined as the need for ETI, switches in non-invasive respiratory support (NIRS), and death under NIRS. We also compared the clinical and blood gas effects at different times.

## Methods

### Study design

This was a single-center retrospective observational study conducted from December 2021 to July 2024, in patients admitted to the Emergency Department (ED) and the Respiratory Intermediate Care Unit (RICU) in the Hospital de Agudos Juan A. Fernández, Buenos Aires, Argentina. The conduct of this study was in accordance with the ethical principles of the Declaration of Helsinki. This study was approved by the institutional review board (code register: 2263) and due to its observational nature, the requirement for informed consent was waived. All patient records and data were anonymized and de-identified prior to analysis.

### Patient screening

We screened patients who received a primary discharge diagnosis of AECOPD. All participants met the COPD criteria established by the Global Initiative for Chronic Obstructive Lung Disease (GOLD) (20), including a forced expiratory volume in 1 s (FEV₁) < 80% and FEV₁/forced vital capacity (FVC) < 70% following bronchodilator inhalation. In cases where COPD diagnosis was suspected without prior spirometry, patients had a documented history of smoking and evidence of emphysema on chest radiograph or CT scan, with no other apparent causes for respiratory acidosis.

General indications for the application of noninvasive respiratory support (NIV or HFNC) in the treatment of AECOPD in our unit include respiratory acidosis (pH ≤ 7.35 and PaCO2 ≥ 50 mmHg), respiratory rate >25 breaths/min, worsening dyspnea with evidence of accessory respiratory muscle use and or persistent hypoxemia despite oxygen therapy.

Eligible patients were adults (>18 years) diagnosed with AECOPD and presenting with mild-to-moderate AHRF, as evidenced by an arterial pH between 7.25–7.35 and a PaCO₂ > 45 mmHg. Exclusion criteria were: (1) severe respiratory failure necessitating immediate endotracheal intubation (ETI), defined as respiratory rate (RR) ≥ 40 breaths/min, severe hypoxemia, severe respiratory acidosis (pH < 7.25), or Glasgow Coma Scale score < 8; (2) contraindications to non-invasive ventilation (NIV) or high-flow nasal cannula (HFNC), including oral/facial trauma or poor expectoration ability; (3) cardiovascular instability requiring vasopressors, acute coronary syndrome, or life-threatening arrhythmias; (4) tracheostomy; (5) cardiac arrest; (6) recent facial or neck trauma, burns, or skin breakdown; (7) pregnancy; or (8) loss to follow-up post-discharge.

### Classification of subjects and NIRS setting

Patients were classified into two groups based on the time from admission to initiation of first-line ventilatory support (either HFNC or NIV). All patients in whom HFNC was started within the first 2 h from admission were included in the HFNC group, if they received at least 2 h of HFNC within the first 24 h. Similarly, patients treated with NIV within the first 2 h of admission were included in the NIV group if they received at least 2 h of NIV within the first 24 h. HFNC was utilized during breaks from NIV without affecting the patient’s original group classification. For instance, a patient initially receiving NIV for 3 h, later transitioned to HFNC or invasive mechanical ventilation (IMV) as rescue therapy, remained in the NIV group.

During treatment, if the patient could not tolerate the assigned treatment, or had respiratory distress, intolerance or carbon dioxide retention unalleviated by assigned treatment, the patient would be changed to the other group treatment modality. These switches were decided by the patient’s attending physician.

### HFNC

HFNC was administered using the AIRVO™ 2 device (Fisher & Paykel Healthcare, Auckland, New Zealand), initially set to a flow rate of 60 L/min at 37°C. If patients reported discomfort, flow and/or temperature were adjusted to achieve the most tolerable setting. The fraction of inspired oxygen (FiO₂) was titrated to maintain peripheral oxygen saturation (SpO₂) between 88 and 92%. Treatment failure of HFNC was defined as a switch to NIV, need for ETI, or death under HFNC therapy.

### NIV

NIV was delivered using the Astral 150 ventilator (ResMed, San Diego, California) with a low-pressure oxygen source and a face mask with a blue elbow (FreeMotion RT041, Fisher & Paykel, Auckland, New Zealand). The ventilator was set to Pressure Support Ventilation (PSV) mode, with positive end-expiratory pressure (PEEP) titrated between 5 and 10 cmH₂O. Pressure support was adjusted to achieve an expiratory tidal volume of 6–8 mL/kg of ideal body weight. FiO₂ was titrated to maintain SpO₂ within the target range of 88–92%. NIV failure was defined as a switch to HFNC, requirement for ETI, or death under NIV therapy.

### Intubation criteria

(1) severe respiratory failure necessitating immediate endotracheal intubation (ETI), defined as respiratory rate (RR) ≥ 40 breaths/min, severe hypoxemia, severe respiratory acidosis (pH < 7.25), or Glasgow Coma Scale score < 8; (2) contraindications to NIV or HFNC, including oral/facial trauma or poor expectoration ability; (3) cardiovascular instability requiring vasopressors, acute coronary syndrome, or life-threatening arrhythmias. These directives are in accordance with the protocols of our hospital and our unit.

### Data collection

For eligible patients, personal characteristics, severity score including the Acute Physiology and Chronic Health Evaluation (APACHE II) and Sequential Organ Failure Assessment (SOFA), relevant comorbidities, Pulmonary function test, GOLD classification, do not intubate order (DNI), and home care settings. We collected vital signs at admission and Arterial Blood Gas (ABG).

### Study endpoints

The primary endpoint was treatment failure, defined as the need for ETI, transition from one non-invasive respiratory support modality to another (e.g., HFNC to NIV or vice-versa), or death under NIRS. The secondary objectives were to evaluate the clinical impact on Respiratory Rate (RR) at different time points (2, 4, 6, and 12 h) as well as changes in baseline ABG and changes at hour 2 and 24. In addition, we measured the duration of NIRS, length of hospital stay and 28-day mortality.

### Statistical analysis

We used data of all available patients without a formal sample size calculation because the purpose of the analysis was to explore the effect of noninvasive respiratory support; we did not specify any *a priori* effect size. Continuous variables are presented as mean and SD (if data were normally distributed) and median and interquartile range (IQR) values (if data were not normally distributed). Categorical variables were described as frequency rates and percentages. Means for continuous variables were compared by paired T-tests or analysis of variance test. Proportions of categorical variables were compared by using the chi-square test or Fisher exact test. Repeated measures analysis of variance, or non-parametric tests of multiple correlated samples (Friedman test for heterogeneity of variance or the skewed distributed data) followed by Bonferroni’s test were performed for the data obtained at multiple time points. Kaplan–Meier curves with the log rank test were used to assess patient survival. *p*-values < 0.05 were considered statistically.

The statistical analysis was performed using R Studio (Version 1.3.1093, R Foundation for Statistical Computing, Vienna, Austria) and Graphpad Prism version 8.0 (Graphpad Software, Inc. La Jolla, CA, United States).

## Results

### Patient characteristics

**Figure 1:**
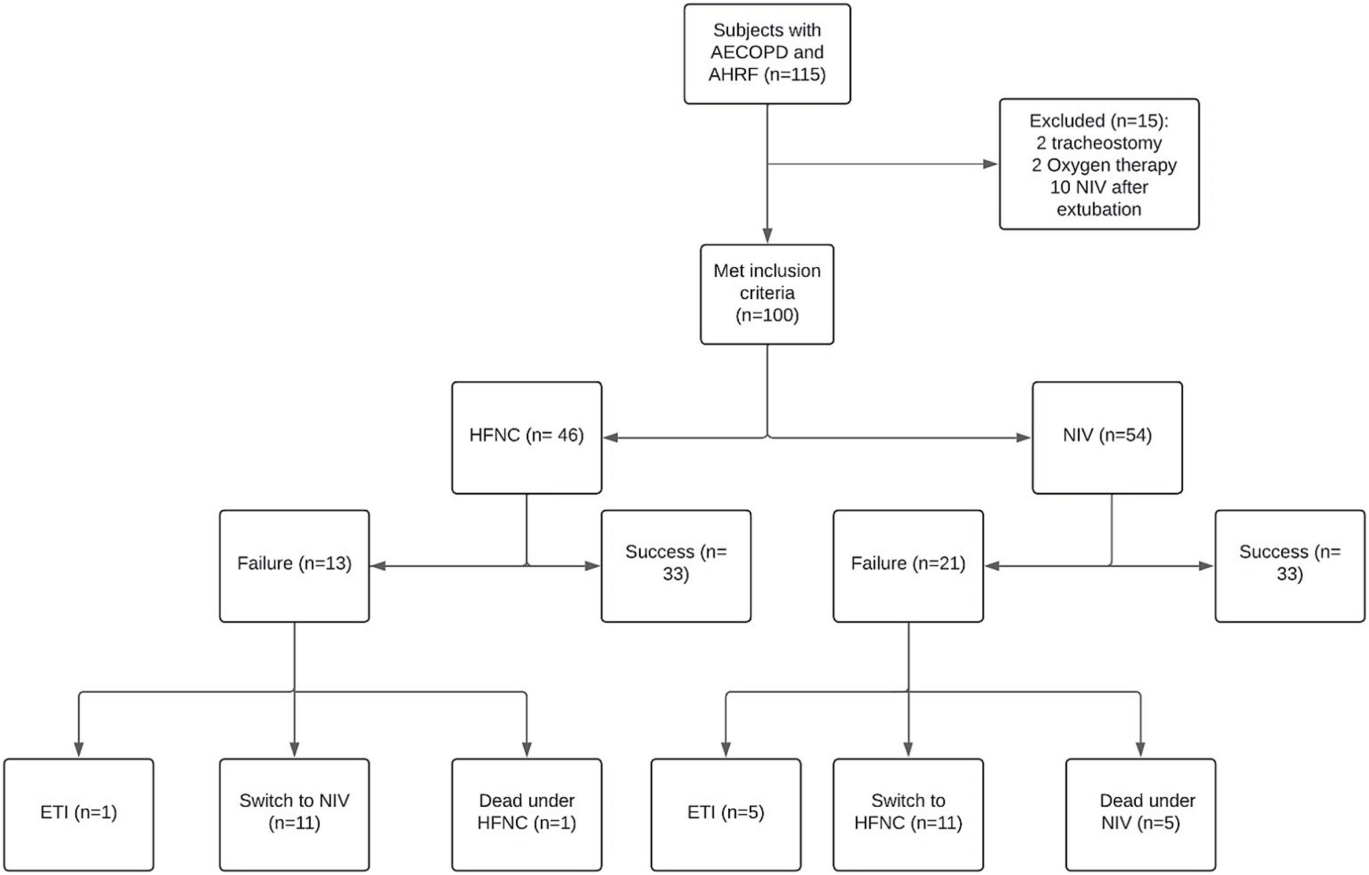
Patients allocation to non-invasive respiratory support. AHRF, Acute Hypercapnic Respiratory Failure; ETI, Endotracheal Intubation; HFNC, High-flow nasal cannula; NIV, Non-invasive ventilation.

Among 115 COPD patients who were admitted to our RICU during the study period. Among these, 15 of them were excluded (2 patients had tracheostomies, 3 require oxygen therapy and 10 had NIV after extubation). One hundred patients were finally selected, including 46 in the HFNC group and 54 in the NIV group (Figure 1). The baseline characteristics of both groups are presented in Table 1. The median (IQR) age of the patients was 70 (65–76) and the majority were male 55%. Forty-five (45%) out of 100 patients were either current or ex-smokers, Congestive Heart Failure (CHF) and hypertension were the most common comorbidities, observed in 30% of patients. Subjects in the NIV group had a significantly higher BMI than the HFNC group 27.34 (24.04–32.44) vs. 21.26 (18.80–30.98); *p* < 0.001, and the presence of CHF was also significant for the NIV vs. HFNC group 39% vs. 20%; *p* = 0.040. Whereas subjects in the HFNC group showed a significant presence of bronchiectasis 22% vs. 7%; p = 0.040. No significant differences were observed in terms of GOLD classification, vital signs and ABG on admission between groups.

**Table 1:** Baseline characteristics of selected patients.

Variables	Total (*n* = 100)	HFNC (*n* = 46)	NIV (*n* = 54)	*p*-value
Female, n (%)	45 (40)	19 (41)	26 (48)	0.492
Age, years	70 (65–76)	70 (65–79)	70 (65–75)	0.643
BMI, kg/m2	25.3 (20.8–31.9)	21.26 (18.80–30.98)	27.34 (24.04–32.44)	<0.001
APACHE II, score	16 (12–20)	15 (12–17)	16 (12–21)	0.110
SOFA, score	3 (2–4)	3 (2–4)	3 (2–4)	0.195
Comorbidities				
Hypertension, n (%)	30 (30)	13 (28)	17 (31)	0.730
Heart Failure, n (%)	30 (30)	9 (20)	21 (39)	0.040
Diabetes mellitus, n (%)	9 (9)	4 (9)	5 (9)	0.920
Chronic kidney disease n (%)	13 (13)	3 (7)	10 (18)	0.080
Bronchiectasis, n (%)	14 (14)	10 (22)	4 (7)	0.040
History of smokers, n (%)	45 (45)	18 (39)	27 (50)	0.280
Obstructive Sleep Apnea, N (%)	7 (7)	0 (0)	7 (0)	0.014
Pulmonary Function Test before an exacerbation (*n* = 51) *				
FEV1, %	49 (32–66)	47 (30–64)	46 (29–63)	0.682
VEF1/FVC, %	47 (35–59)	46 (34–58)	45 (33–57)	0.806
GOLD Class, n (%)				
I	6 (6)	4 (9)	2 (4)	0.721
II	20 (20)	8 (17)	12 (22)	
III	50 (50)	23 (50)	27 (50)	
IV	24 (24)	11 (24)	13 (24)	
Do not intubation order, n (%)	15 (15)	6 (13)	9 (17)	0.610
Home care				
LTO, n (%)	30 (30)	17 (36)	13 (24)	N/A
NIV, n (%)	14 (14)	5 (10)	9 (16)	N/A
HFNC, n (%)	3 (3)	3 (7)	0 (0)	N/A
Vital signs at admission				
Respiratory Rate, breath/min	30 (28–34)	30 (28–34)	31 (30–33)	0.730
Heart Rate, beats/min	98 (89–105)	99 (90–110)	97 (89–102)	0.260
SpO2, %	89 (87–92)	90 (88–93)	89 (86–92)	0.142
Arterial Blood gases at admission				
pH	7.31 (7.29–7.33)	7.31 (7.30–7.33)	7.31 (7.28–7.33)	0.439
PaCO2, mmHg	62 (58–67)	62 (58–65)	63 (58–68)	0.460
PaO2, mmHg	63 (57–69)	62 (55–68)	64 (59–69)	0.394
HCO3 − (mmol L − 1)	29.4 (26.7–32.7)	28.7 (26.6–31.7)	29.9 (27.5–33.0)	0.204
PaO2/FiO2	227 (163–281)	228 (165–299)	218 (163–271)	0.420

Data are shown as mean ± standard deviation, number (%) patients, or median (interquartile range). HFNC, High-Flow Nasal Cannula; NIV, Non-Invasive Ventilation; BMI, Body Mass Index; APACHE, Acute Physiology and Chronic Health Evaluation; SOFA, Sepsis related Organ Failure Assessment; GOLD, Global Obstructive Lung Disease; LTO, Long-Term Oxygen; PaCO2, Partial pressure of arterial carbon dioxide; PaO2, Partial pressure of arterial oxygen; HCO3−, Bicarbonate; FiO2, Fraction of inspired oxygen. * 27 case in the HFNC group and 24 in the NIV group.

### Non-invasive respiratory support setting

The initial FiO2 in the HFNC group was 0.30 (0.25–0.40) %, and the gas flow rate was 60 (40–60) L/min. While initial FiO2 in the NIV group was 0.25 (0.25–0.30) %, PSV 10 (8–12) cmH2O, PEEP 9 (8–10) cmH2O.

### Clinical outcomes

Regarding the primary outcome of the study, no significant differences were found between groups in terms of failure (Table 2), and in relation to the reasons for these failures such as intubation, switch in treatments or death under NIRS, no significant differences were found as well. Secondary outcomes of treatment switch were intolerance in the NIV group (21% vs. 0%; *p* < 0.001). Subjects who were started on HFNC and switched to NIV for a worsening of respiratory distress, 6 (15%) vs. 0; *p* = 0.020 and worsening of carbon dioxide retention in 5 (11%) vs. 0: *p* = 0.013 (Table 3). The NIV group changed treatment significantly earlier (2.84 [2.45–3.25]) vs. (6.00 [4.00–6.00]; *p* = 0.002) hours than the HFNC group.

**Table 2:** Primary endpoint and reason of failure.

Variables	Total (*n* = 100)	HFNC (*n* = 46)	NIV (*n* = 54)	*p*-value
Treatment failure, *n*, %	34 (34)	13 (28)	21 (39)	0.263
Reasons of failure				
Intubation, *n*, %	6 (6)	1 (2)	5 (9)	0.137
Treatment Switch, *n*, %	22 (22)	11 (24)	11 (20)	0.670
Dead under NIRS, *n* (%)	6 (6)	1 (2)	5 (9)	0.140

HFNC, High-Flow Nasal Cannula; NIV, Non-Invasive Ventilation; NIRS, Non-invasive respiratory support.

**Table 3:** Analysis of treatment failure in the HFNC and NIV groups.

	HFNC (*n* = 46)	NIV (*n* = 54)	*p*-value
Treatment intolerance, *n* (%)	0	11 (21)	<0.001
Aggravation of respiratory distress, *n* (%)	6 (15)	0 (0)	0.020
Aggravation of carbon dioxide retention, *n* (%)	5 (11)	0 (0)	0.013

HFNC, High-Flow Nasal Cannula; NIV, Non-Invasive Ventilation.

**Figure 2:**
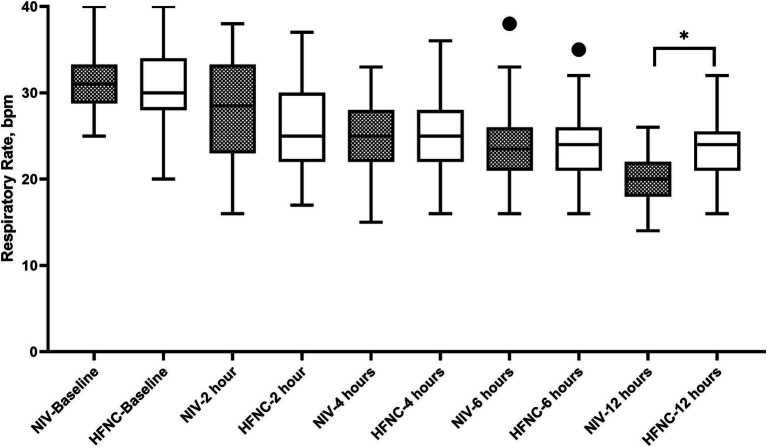
Respiratory rate during non-invasive respiratory support. HFNC, High-flow nasal cannula; NIV, Non-invasive ventilation.

**Figure 3:**
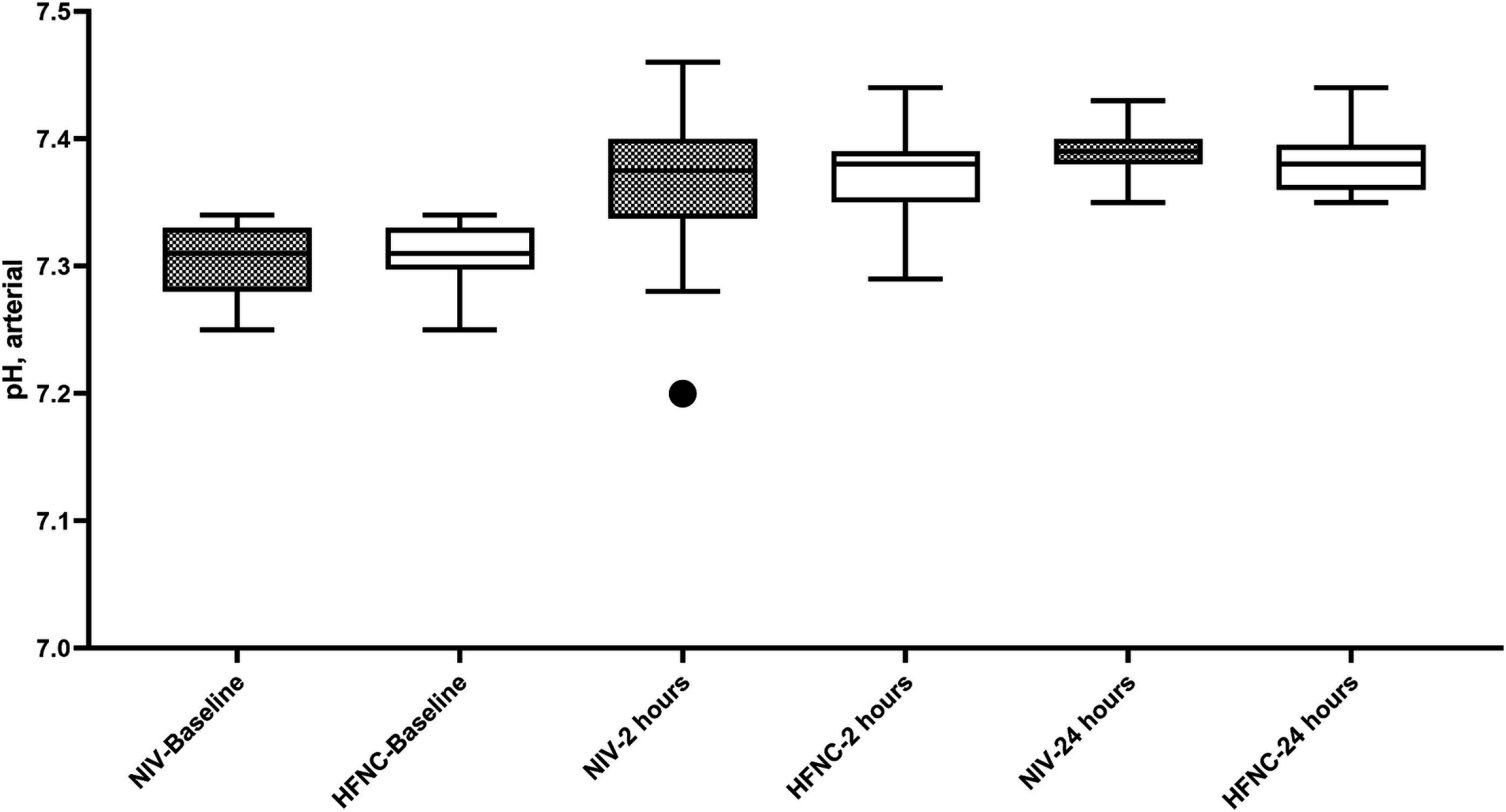
pH during Non-Invasive Respiratory Support. HFNC, High-flow nasal cannula; NIV, Non-invasive ventilation.

**Figure 4:**
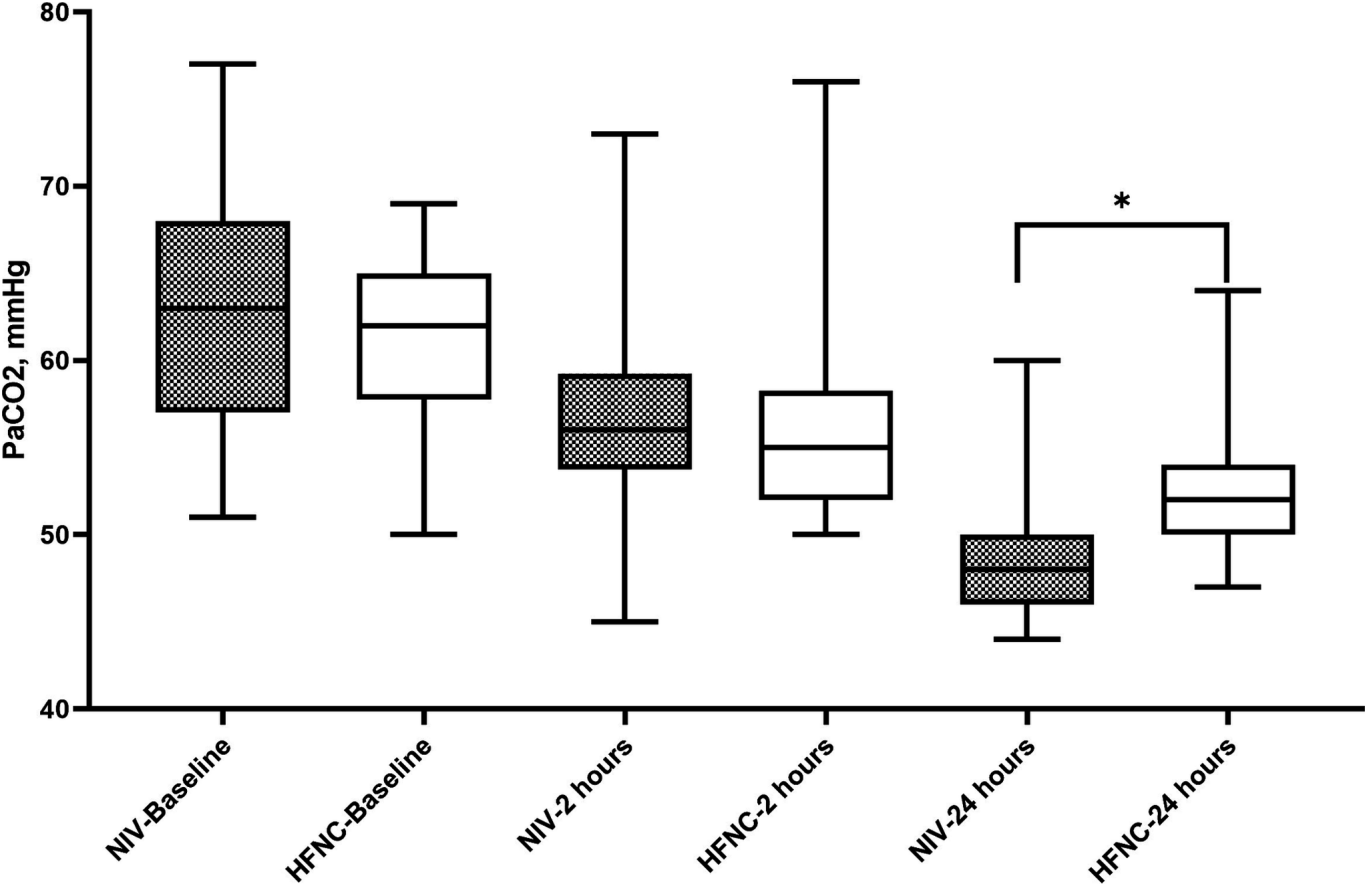
Partial pressure of carbon dioxide during non-invasive respiratory support. HFNC, High-flow nasal cannula; NIV: Non-invasive ventilation.

**Figure 5:**
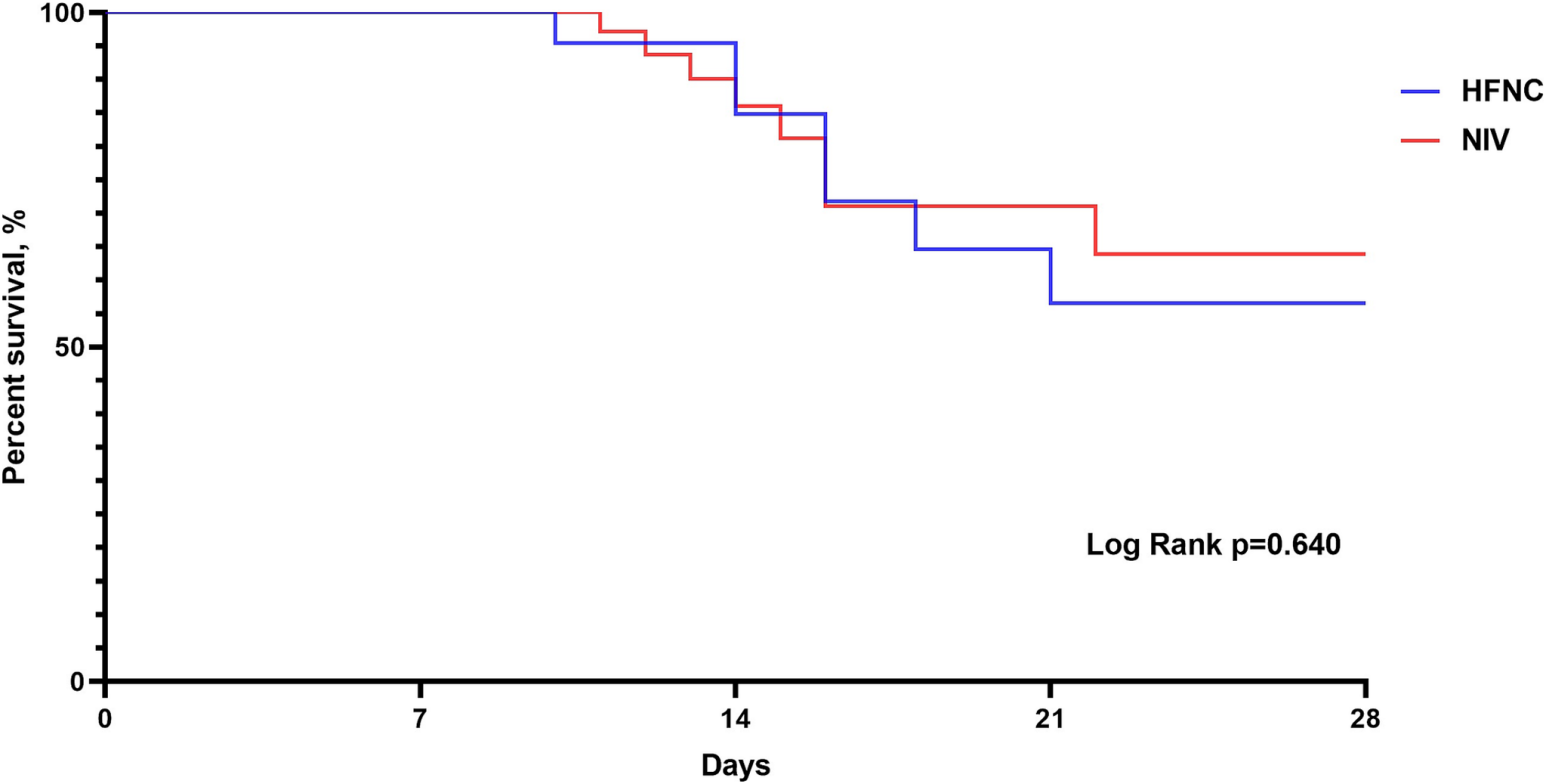
Kaplan–Meier curve analysis for 28-d mortality. HFNC, High-flow nasal cannula; NIV: Non-invasive ventilation.

Regarding RR, we did not observe significant differences at baseline, but we did find that at 12 h of treatment the NIV group had a lower RR than the HFNC group, 20 (19–22) vs. 23 (19–25); *p* = 0.015, respectively. During the 2, 4, 6 and 12 h, both treatments reduced RR from baseline (Figure 2). ABG improved from baseline in both groups at 2 and 24 h of treatment, with no significant differences in pH at 2 and 24 h between groups (Figure 3), although we report a significant decrease in PaCO2 in the NIV group versus the HFNC group 49 (46–52) vs. 52 (49–53); *p* = 0.010 (Figure 4) at 24-h. There was no significant difference in the duration of NIRS; HFNC 7 (3–13) days and NIV 8 (4–12) days, and no significant difference in length of hospital stay; HFNC 9 (7–16) days and NIV 12 (8–16) days. Overall treatment failure in our study was 34% (34/100) and total mortality at 28 days was 15% (15 of 100 patients) p-Log Rank; *p* = 0.640 (Figure 5).

## Discussion

In this retrospective study we compared the use of NIV or HFNC as first-line treatment during AECOPD. We found (1) Patients had similar respiratory variables at admission, regardless of the type of support used. There was no significant difference in treatment failure defined as, requirement for ETI, switch from one non-invasive respiratory support to another or death under NIRS. (2) We observed that subjects who had to switch from NIV to HFNC did so mainly because of interface intolerance, and this change occurred earlier than in the HFNC group. (3) Subjects with HFNC were switched to NIV for respiratory distress or increased PaCO2. (4) We report a significant improvement at 12 h in RR reduction in the NIV group. (5) At 24 h, the NIV group demonstrated a significantly lower PaCO2 than HFNC group.

In our study, requirement for IMV was low, and these results are in line with those reported by a retrospective, propensity score match study published by Wang et al. where the authors report a failure rate of 4.5% in the HFNC group vs. 11.4% in the NIV group (21). In other studies, the percentage of failure requiring ETI in both groups ranges between 20 and 27%, with no significant differences between the two groups in this outcome (16, 17, 22). In contrast to these results, a recently published RCT by Tan et al. reported higher failures in the HFNC vs. NIV group (14.2 vs. 5.4%). This non-inferiority RCT showed that HFNC was not shown to be non-inferior to NIV for preventing treatment failure for AECOPD patients with moderate AHRF, in both an intention-to-treat analysis or in a per-protocol analysis (19). In contrast to Tan et al.’s study, which used a fixed initial HFNC flow rate of 40 L/min, our study utilized a initial flow rate of 60 L/min. Whether this difference in initial flow rate influenced carbon dioxide removal or other outcomes is uncertain and warrants further investigation. Treatment failure defined as a switch of treatment, i.e., HFNC switch to NIV or vice versa seems to be a frequent practice in this setting. If analyzing the reasons for a switch from HFNC to NIV, we found that 32% of subjects in RCT published by Cortegiani et al. (18) required a switch to NIV within 6 h of HFNC treatment. These changes may be due to poor response in PaCO2 reduction or worsening respiratory distress (18). However, the authors themselves acknowledge that their results may be influenced by variations in expertise, settings, and protocols across participating centers. Furthermore, they emphasize that their findings may not be generalizable to settings with different levels of monitoring and care. Sun et al. reported a 7.7% switch from HFNC to NIV (17), and Tan et al. (19) 11.5%. Consistent with these results, our study had a 24% switch from HFNC to NIV and 20% from NIV to HFNC. While HFNC was successful as an initial therapy in the majority of patients, our study revealed a 13% (*n* = 28) failure rate. Importantly, 85% (n = 24) of those who failed HFNC required subsequent NIV. The reasons for switching from HFNC to NIV were marked by worsening PaCO2 or respiratory distress. This finding underscores the clinical significance of developing tools to aid in the timely and effective selection of appropriate respiratory support strategies, ensuring that patients receive the therapy best suited to their individual needs. In both studies there were no significant differences in terms of change of treatment or reason. Consistent with Cortegiani, a higher proportion of patients in the NIV groups showed poor tolerance to the intervention at 6-h (74%) compared to HFNC (35%) (*p* = 0.0019) (18). In our study, NIV was less tolerated (21 vs. 0%) vs. HFNC. While the results showed that obesity was significantly associated with the need for NIV (Table 4), the potential for selection bias should be considered when interpreting the causality of this relationship.

**Table 4:** Multivariate logistic regression analyses were performed to identify factors related to the requirement for NIV.

Variable	OR (multivariable)	*p*-value
BMI	1.09 (1.02–1.18)	0.013
CHF	0.5 (0.17–1.42)	0.197
Bronchiectasis	0.4 (0.20–1.41)	0.168

BMI, Body Mass Index; CHF, Chronic Heart Failure.

In terms of mortality, the studies showed no significant differences between treatments. In line with our results, Sun et al. reported a 28-d mortality in the HFNC group 15.4 vs. 14% in the NIV group (17). In another study Lee et al. showed a 30-d mortality in the HFNC group of 15.9 vs. 18.2 in the NIV group (16). Similar to our results no significant differences in 28-d mortality were found. Mortality in this setting varies between 5 and 18%.

The clinical effectiveness of both NIRS in reducing RR was significant from baseline with no significant differences at 2, 4 and 6 h. Tan et al., in their study, reported no significant differences at 1 and 12 h of treatment (19). In contrast to our study, the NIV group showed a lower RR at 12 h. This result could be due to the application of HFNC during NIV breaks. These treatments in combination could have an effect on improving RR. It is known that the application of conventional oxygen therapy during NIV breaks could lead to increased work of breathing (12). In terms of gas exchange, PaCO2 at 24 h in the NIV group was lower, this can also be explained by the use of HNFC during NIV breaks.

In our patient cohort, 45% had a history of smoking (Table 1). This may partially account for the findings, as in our country and region, risk factors such as biomass fuel exposure, passive smoking, and advancing age significantly contribute to COPD development (23). Although this study does not specifically examine these risk factors and data are limited, they warrant further investigation in future research. Additionally, 49% (*n* = 51) of patients had undergone pulmonary function testing prior to admission. Due to the retrospective design, some PFT results are unavailable, and diagnoses were based on medical record documentation.

This study has some limitations. First, in this retrospective study, the decision to start HFNC or NIV was made on a clinical basis, making it susceptible to selection bias. For COPD patients with moderate AHRF in our unit, both HFNC and NIV were the first-line choice for treatment, which could reduce selection bias for other ventilation devices. Second, because the sample size is relatively small, the risk factors for treatment failure of HFNC were not analyzed.

Third, we did not exclude subjects with NIV at home or those who had a do not intubate order, as these could have increased the number of treatment failures due to advanced disease.

Fourthly, since patients may switch from HFNC to NIV to due to worsening (respiratory distress or carbon dioxide retention), but from NIV to HFNC due to improvement or intolerance, it seems desirable that future studies take into account that these patients may be very different from each other, even if they follow the same treatment. This approach could be useful in interpreting the results and understanding the association between reasons for treatment failure and outcomes irrespective of the support used.

Finally, our findings should be interpreted with caution, as we did not collect data on patient-reported outcomes such as comfort and dyspnea. The absence of these measures may introduce bias, as clinicians’ perceptions of patient comfort could have influenced treatment decisions. Future studies should include these important subjective outcomes. Furthermore, the decision to switch between HFNC and NIV in this study was made by the attending physician, inevitably introducing a degree of subjectivity that reflects real-world clinical practice.

Further studies are still needed to determine patterns of alarm in subjects with AECOPD using HFNC as first-line treatment.

## Conclusion

In subjects with AECOPD and AHRF, the utilization of HFNC compared to NIV did not demonstrate increased failure rates. HFNC exhibited superior tolerance relative to NIV and serves as a viable alternative for patients who cannot tolerate NIV. Furthermore, employing HFNC during NIV breaks may improve RR and arterial carbon dioxide pressure levels. The dynamic interplay between HFNC and NIV, driven by clinical responses—transitioning to NIV for respiratory deterioration or carbon dioxide retention and reverting to HFNC for improvement or intolerance—underscores the importance of stratifying patient populations in future research. Recognizing the distinct clinical trajectories, despite a common treatment modality, may contribute to more accurate interpretation of outcomes and enhance understanding of the relationship between the underlying causes of treatment failure and corresponding clinical endpoints.

## Data Availability

The underlying raw data supporting the conclusions of this article will be available from the authors. Further inquiries can be directed to the corresponding author.
